# Wide dynamic range enrichment method of semiconducting single-walled carbon nanotubes with weak field centrifugation

**DOI:** 10.1038/srep44812

**Published:** 2017-03-20

**Authors:** Wieland G. Reis, Željko Tomović, R. Thomas Weitz, Ralph Krupke, Jules Mikhael

**Affiliations:** 1 Carbon Materials Innovation Center (CMIC), BASF SE, 67056 Ludwigshafen, Germany; 2 Physics of Nanosystems, Physics Department, NanoSystems Initiative Munich and Center for NanoScience (CeNS) Ludwig Maximilians Universität München, Amalienstrasse 54, 80799 Munich (Germany); 3 Department of Materials and Earth Sciences, Technische Universität Darmstadt, 64287 Darmstadt, Germany; 4 Material Physics Research, BASF SE, 67056 Ludwigshafen, Germany

## Abstract

The potential of single–walled carbon nanotubes (SWCNTs) to outperform silicon in electronic application was finally enabled through selective separation of semiconducting nanotubes from the as-synthesized statistical mix with polymeric dispersants. Such separation methods provide typically high semiconducting purity samples with narrow diameter distribution, i.e. almost single chiralities. But for a wide range of applications high purity mixtures of small and large diameters are sufficient or even required. Here we proof that weak field centrifugation is a diameter independent method for enrichment of semiconducting nanotubes. We show that the non-selective and strong adsorption of polyarylether dispersants on nanostructured carbon surfaces enables simple separation of diverse raw materials with different SWCNT diameter. In addition and for the first time, we demonstrate that increased temperature enables higher purity separation. Furthermore we show that the mode of action behind this electronic enrichment is strongly connected to both colloidal stability and protonation. By giving simple access to electronically sorted SWCNTs of any diameter, the wide dynamic range of weak field centrifugation can provide economical relevance to SWCNTs.

A series of unique properties suggests carbon nanotubes as material of the future. The exceptional electronic properties have made especially single-walled carbon nanotubes (SWCNTs) popular among researchers for numerous applications^1^,^2^,^3^.

While numerous multi-walled carbon nanotubes enhanced products are commercially available (i.e. Ultraform^®^ N2320 C, BASF), electronic devices with SWCNTs as main building block did not make it into the market yet. Probably one of the main technological hurdles for SWCNTs are their significant structural heterogeneities in the available materials on the market. Well established synthesis processes for single-walled carbon nanotubes (SWCNTs) are known to result in strong polydispersities in terms of length, diameter, chiral angle or even presence of batch dependent contents of amorphous carbon or catalyst^4^,^5^,^6^,^7^,^8^,^9^,^10^,^11^. Unfortunately, the more recently developed production routes^12^,^13^,^14^,^15^ yielding narrow chirality distributions still suffer from limited scalability and often insufficient purity.

For electronic applications, however, the premise is to have high purity fractions of electronically sorted SWCNTs^16^,^17^,^18^,^19^. While specific applications, such as integrated circuits^20^ or photothermal tumor elimination in biomedicine^21^, tend to use single-chirality semiconducting SWCNTs, many other applications only require a mixture of high purity semiconducting tubes. Transparent solar cells, for example, require small diameter polychiral semiconducting SWCNTs to efficiently harvest light^22^,^23^. For such applications, scalable and high yield methods able to enrich mixtures of semiconducting SWCNTs with small or large diameters are needed.

Using amphiphilic and polymeric surface actives, the as-synthesized powder of SWCNTs is typically dispersed in organic or aqueous solvents^24^. This post synthesis treatment is usually needed to simplify the handling of such toxic nanomaterial and to suppress the bundling and agglomeration of the tubes that hold a strong hydrophobic character.

Previous investigations have shown that polymeric dispersants, such as the Pluronics or Tetronics, also allow an electronic sorting and enrichment using for e.g. density gradients ultracentrifugation^25^,^26^. However these polymers do not seem to be able to disperse certain diameters^25^. Subsequently, the separation using these polymers works only for selected SWCNT sorts, i.e. stemming from specific synthesis routes. In other words, such separation methods are limited not only by the use of expensive and not scalable lab equipment (i.e. ultracentrifuges) but also have a narrow dynamic range with respect to tube diameters^27^,^28^.

Conjugated polymers have enabled simple access to single-chirality fractions of SWCNTs^29^,^30^,^31^,^32^,^33^. Actually, distinct conjugated polymers have shown to be able to selectively disperse semiconducting chiralities of SWCNTs^19^. However, this technology delivers typically low yields since most polymers are only suitable to disperse nanotubes of certain diameters and even preferring single chiralities within a given diameter distribution of semiconducting nanotubes^33^,^34^. Thus the selective dispersion of semiconducting SWCNTs with one polymer excludes a wide range of semiconducting SWCNTs limiting the yield.

In a recent publication a novel semiconducting enrichment of SWCNTs using only weak centrifugal fields was introduced^35^. Here, a polyarylether (PAE) polymeric dispersants with strong amphiphilic character was combined with a low viscosity heavy material, Sodium Polytungstate (SPT) at low pH. In one single separation step, metallic SWCNTs and large impurities were excluded from an aqueous dispersion.

In this publication we demonstrate that due to its unique separation mechanism, the weak field centrifugation (WFC) method is a separation technology with a broad dynamic range. Not only semiconducting HiPco SWCNTs having a mean diameter of about 1 nm can be enriched. Plasma Torch and Arc Discharge semiconducting SWCNT s with diameters ranging from 1.0 to 1.8 nm can also be enriched in one single step using the same polymeric dispersant and low centrifugal fields. To our knowledge, no other sorting process for pristine electronic type separation has shown applicability independent of the raw SWCNT material while being as simple as the water based WFC. In fact, we first demonstrate that the PAE dispersant is able to adsorb and disperse different SWCNT sorts and diameters. We also investigate the influence of temperature while dispersing the carbon material and during separation. In contrast to other separation methods also using centrifugal fields^26^, we show here that for WFC higher temperatures lead to an increase in semiconducting purity. This proofs the difference in the separation mechanism of WFC that is mainly based on a selective pH-dependent colloidal stability.

## Results and Discussion

Enrichment of semiconducting SWCNTs using only weak centrifugal fields (<10.000 × *g*) in aqueous environment requires a tailored comb polymer with strong amphiphilic properties (see chemical structure in Supporting Information)^35^. In order to investigate the ability of such polymer to first disperse and then enrich semiconducting SWCNTs stemming from different synthesis routes, i.e. having different diameters, we first investigate its adsorption on nanostructured carbon materials.

**Figure 1:**
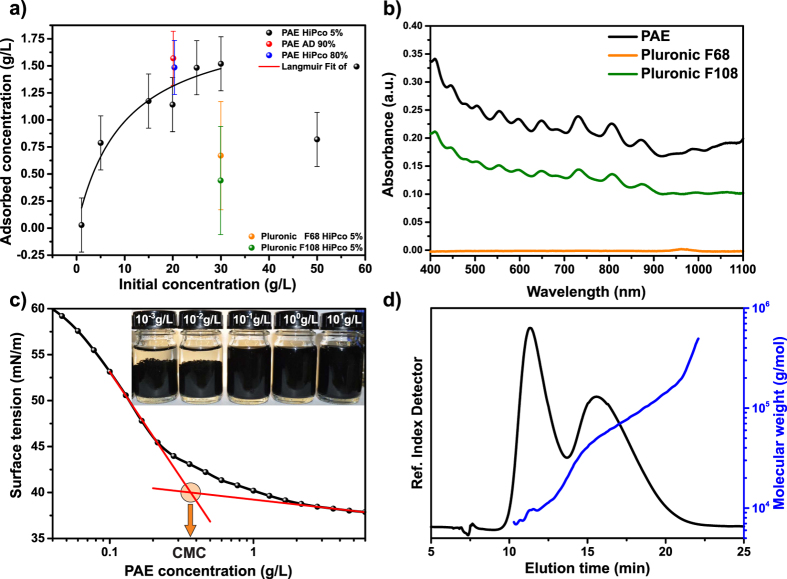
(**a**) Measured adsorption isotherm (black dots) and Langmuir fit (black line) of PAE on 2.5 wt% HiPco material with 5 wt% SWCNT content at pH 7. Single point measurements of PAE adsorption on Arc Discharge and HiPco with 90 wt% and 80 wt% SWCNT content, respectively (blue & red dots). Single point measurement of Pluronic F68 and F108 adsorption on HiPco with 5 wt% SWCNT content are also shown (green & orange dots). (**b**) Absorbance spectra of dispersed SWCNT content separated from raw dispersions of 0.5 wt% HiPco powder in aqueous 2 wt% of various polymeric dispersants. (**c**) Surface tension measurements of aqueous PAE solutions and determination of the critical micelle concentration (CMC) at 23 °C. Inset: picture showing the colloidal stability of HiPco material with 5 wt% SWCNT content at increased PAE Concentration. (**d**) Chromatographic analysis of PAE. In black, the elution of PAE polymer as measured with asymmetrical-field field flow fractionation with differential refractive index detector. In blue the corresponding molecular weight determined with a multi angle light scattering detector.

In Fig. 1a the adsorption of a PAE polymer, set at different concentrations, on HiPco raw material with a 5% SWCNTs content (as delivered) is presented. The adsorbed polymer amount on 25 g/L raw material was determined by measuring the amount of free polymer using absorption spectra or a refractometer (see Supporting Information).

At low polymer concentrations, a very steep increase in adsorption can be seen. At concentrations above 15 g/L, the strong increase is greatly reduced and the amount of adsorbed polymer remains almost constant. For an initial polymer concentration of about 50 g/L a decrease in the adsorption was measured. Clearly, at higher amounts of polymer the carbon surface is saturated that is why a plateau region is detected. The decrease in adsorption at extremely high concentrations is known in literature and is not specific to this system^36^. The pre-plateau adsorption data can be fit with a Langmuir isotherm (black line) and is added here as a guide to the eye. This good fit might also suggest that the PAE adsorbs as a single layer as long as free surface sites are available.

In order to evaluate the influence of the SWCNT content, adsorption at higher initial PAE concentration (20 g/L) on materials with higher SWCNT content was also measured. Also here a concentration of 25 g/L was used but for raw SWCNTs from HiPco materials and arc discharge containing 80 wt% and 90 wt% of SWCNTs, respectively. Although one would expect differences in the total surface specific area between the low and high SWCNT content samples, the adsorption on both samples is comparable to the adsorption on the material with only 5 wt% SWCNT content. This shows that the adsorption of the PAE polymer is not strongly dependent on the carbon morphology nor on the SWCNT mean diameter. Apparently, the PAE dispersant does not discriminate between differently structured carbon nano-surfaces at neutral pH.

For a polymer concentration of 30 g/L we also tested the adsorption of two Pluronic polymers (Pluronic F68 & F108). Both of these polymers were reported to be suitable for dispersing and separating SWCNT by electronic type using DGU^25^. For both polymers the adsorption was found to be significantly lower, by a factor greater than 2, compared to the adsorption of the PAE polymer. This proofs the strong affinity of the PAE to carbon surfaces.

However, adsorption does not automatically imply individualization and stabilization of SWCNTs. Strong electrosteric repulsion is usually needed to overcome the attractive Van-der-Waals forces. To review the degree of individualized nanotube stabilization, a polymer concentration of 20 g/L was used to disperse a concentration of 5 g/L of raw HiPco SWCNT material with 5% SWCNTs content. The stabilized and individualized SWCNT contents were isolated by submitting the dispersions to density gradient ultracentrifugation (see Supporting Information). Figure 1b shows the absorbance spectra of the SWCNT contents in each dispersion created by the addition of a specific polymeric dispersants. Clearly, the PAE polymer is able to stabilize the highest amount of individualized SWCNTs in the dispersion. Pluronic F108 also stabilizes HiPco SWCNTs, however, Pluronic F68 is not suitable for stabilization of the small diameter HiPco SWCNTs (0.7–1.2 nm) in contrast to stabilizing larger diameter SWCNTs (1.2–1.8 nm) as reported elsewhere^25^,^26^.

Colloidal stability of a dispersion can be strongly depending on the critical micelle concentration (CMC) of a dispersant. Surpassing the CMC improves for many surfactants the stabilization of SWCNTs in water^37^. The CMC of polymeric surface actives can be measured using surface tension analysis. Figure 1c shows the surface tension dependency on the PAE concentration in an aqueous solution. At low concentrations, a fast decrease in the surface tension can be seen. At higher concentrations, a second regime where the decrease is slower can also be detected. Out of the intersection of interpolated linear fits to both regimes, the CMC value was determined to be about 0.370 ± 0.02 g/L. Clearly, also for PAE dispersants, stable SWCNT dispersions require concentrations above the CMC. This can be seen in the inset in Fig. 1c. Here, PAE stabilized dispersions of HiPco raw material with 5% SWCNT content with polymer concentrations ranging from 10^−3^ to 10^1^ g/L are shown. For concentrations well below the CMC, a sediment can be clearly observed. Absorption spectra of the according supernatants after centrifugation for 30 min at 250.000 × *g* further reveal that effective dispersing of SWCNTs requires concentrations above 0.1 g/L (see Supporting Information).

Polymerization reactions typically lead to polydispersity in the molecular structure of the final reaction product^38^. The synthesis of the PAE polymer was described in detail in a previous work^35^. Here, we study the molecular weight distribution of the PAE Polymer using a powerful fractionating technique called Asymmetrical-Field Field Flow Fractionation (AF4). AF4 is able to analyse not only solution polymers but also their tendency to form larger structures such as micelle or aggregates. Figure 1d shows the bimodal elution as detected using a differential refractive index detector after fractionation. At low elution time, low molecular weight polymers are detected. At high elution time, larger structures are detected. The molecular weight vs. time, plotted in Fig. 1d, shows that a PAE solution contains solution polymers with an average molecular weight of about 9,000 g/mol and about 55 wt% of micelles with an average molecular weight of about 66,000 g/mol. Using a Quasi elastic light scattering detector (QELS) we also determine the hydrodynamic radius (R_h_) of the polymers and micelles at peak maximum. The data analysis shows that the solution polymers have a radius of about 2** **nm while the hydrodynamic radius of the micelles is almost twice as large.

In order to demonstrate the wide dynamic range of WFC, i.e. its ability to separate and enrich semiconducting SWCNTs with different diameters, we selected three sorts of SWCNT powders: HiPco, Plasma Torch (PT) and Arc Discharge (AD). The PT and AD processes lead to the production of larger diameter SWCNTs (1.0–1.8 nm) while HiPco process gives diameters ranging from 0.7 to 1.2 nm^39^,^40^ (See Supporting Information for spectra of the dispersed SWCNTs). Knowing that the separation mechanism in WFC mainly relies on selective stability in acidic environment^35^, also here we tested the influence of acidity of the heavy liquid used for separation.

**Figure 2:**
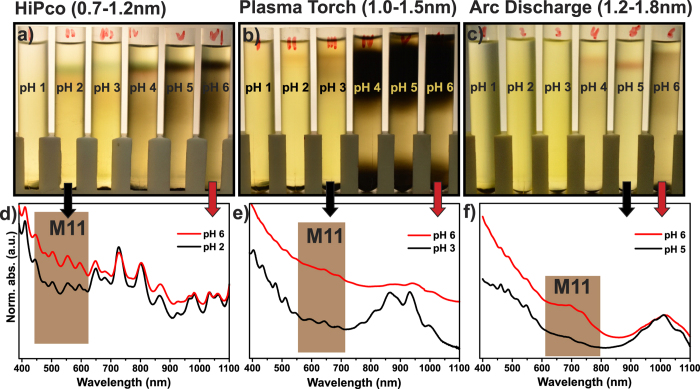
Separation of (**a**) HiPco SWCNTs with mean diameter 1.0 nm (**b**) PT SWCNTs with mean diameter 1.2 nm and (**c**) AD SWCNTs with mean diameter 1.5 nm using weak field centrifugation (WFC) at 10.000 × *g* for 18 h. The pH value in the tubes increases from left to right (from pH 1 to pH 6). (**d**,**e** and **f**) Absorbance spectra of separated fractions from the upper part of tubes for each SWCNT sample.

In Fig. 2(a,b,c) the separation state after a single step WFC, i.e. without any pre-purification, is shown. We performed the experiment by superimposing a volume of 0.5 ml of a nanotube dispersion (0.5 wt% raw CNTs, 2 wt% PAE) set at pH 4 onto a SPT column (~4 ml) having pH values ranging from 1 to 6. The WFC run was set at 10.000 × *g* for 18 h, while stronger centrifugal fields generally decrease the time to reach the separation state.

It is important to note that the three selected samples do not only differ in the diameter distribution but also in the SWCNT content. The HiPco and PT samples have a large non-nanotube content of about 95 wt% and 70 wt%, respectively, where the raw HiPco material contains an unknown percentage of ethanol. For the AD sample, the non-nanotube content was only 10 wt%. The impact of the impurities is observable as wider and darker banding in Fig. 2 at higher pH.

The same qualitative separation behaviour is observed for all three SWCNT sorts. Independent of nanotube diameter and amorphous carbon content, at extremely low pH values all the carbon material is lost towards the bottom of tube. Similar to what was reported previously for HiPco nanotubes^35^ also for the large diameter SWCNTs, at intermediate acidity, banding is observed. Close to neutral conditions, no separation is observed. In order to demonstrate that electronic type separation is obtained, selected UV-vis-NIR spectra of sorted and unsorted samples are plotted in Fig. 2(d,e,f). Independent of tube diameter, the absorbance of the first order metallic (M11) transition region is reduced at intermediate pH values. This proofs that WFC can achieve electronic type separation using the same polymer for different SWCNT sorts.

In Fig. 2(d,e,f) it is also noticeable that for large nanotube diameter, the banding is observed at slightly higher pH values. Apparently, to achieve electronic type enrichment, pH values of about 2 to 3 are required for small diameter distributions (HiPco & PT) and pH values of about 4 to 5 for the large diameter distribution (AD). It appears that the larger the mean diameter of the nanotubes is, the less acidic the environment has to be in order to reach separation. Moreover, for HiPco nanotubes stability is completely lost at pH 1, while for PT nanotubes the stability is widely lost up to pH 2. The larger diameter AD tubes lost their stability up to pH 3. This behaviour could be explained by the fact that larger diameter semiconducting nanotubes possess smaller band gaps, which makes them prone for protonation in less acidic environment^41^. Protonation is expected to result in a negative image charge on the SWCNTs that adversely affects the SWCNT/PAE interaction^41^. Subsequently, higher pH values are required to reach a regime where the stability of the semiconducting SWCNTS is not lost. Metallic SWCNTs possess no band gap and should be protonated regardless of their diameter.

Out of the diameter dependent pH shift, one could conclude that semiconducting enrichment in WFC is mainly driven by the strength of protonation. To better understand the separation mechanism, we performed further experiments where the centrifugation temperature was varied. As a matter of fact, decreasing temperature allows a higher maximum content of oxygen in aqueous systems^42^. The presence of dissolved oxygen in aqueous systems strongly influences protonation^41^ and therefore decreasing temperature favours protonation of SWCNTS^41^. Homenick *et al*. have already reported temperature dependent DGU separation experiments^26^, in which lowering temperature has resulted in higher purity enrichment of semiconducting SWCNTs.

The temperature dependent separation experiment was conducted at a fixed g-force of about 10.000 × *g*, the HiPco carbon dispersion was set to pH 4 and the SPT concentration of 26 wt% was set to pH 2. The temperature was controlled between 10 °C and 35 °C in steps of 5 °C.

**Figure 3:**
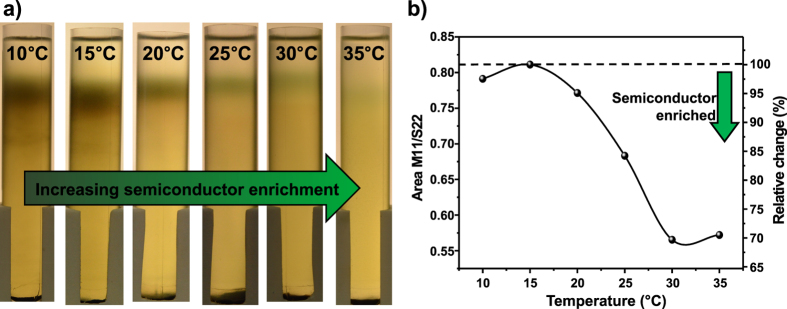
(**a**) Separation of HiPco SWCNTs was performed at different temperatures using weak field centrifugation (10.00 × *g)* for 18 h. The temperature in the tubes increases from left to right (from 10 °C to 35 °C). (**b**) The nanotube fractions from the upper part of the tubes were analysed using normalized (peak 727 nm) UV-vis-NIR spectra. The area ratio between the M11 transitions and S22 transitions was formed and is plotted here as a function of temperature.

The resulting separation state after 18 hours of centrifugation is illustrated in Fig. 3. Surprisingly, the banding at the upper part of the tube is gradually lost for lower temperatures. Clearly, the separation mechanism in WFC is hindered at low temperatures. Increased temperatures actually result in better purity for the semiconducting fraction of SWCNTs.

It is also interesting to note the broadening of the upper band at higher temperatures, especially at 35 °C. This could be explained by the change of the SPT viscosity. The temperature increase has in fact an attenuating influence on the viscosity^43^. This is then expected to direct the density barrier that is maintained at such low centrifugal fields^35^ into faster diffusive softening. Therefore a broader spatial distribution of the SWCNTs across their isopycnic points is induced^44^.

In order to quantify the temperature effect on the semiconductor enrichment, UV-vis-NIR spectra (Supporting Information) of the SWCNT fractions extracted from the upper part of the tube, were recorded. Subsequently, the ratio of the area under the M11 (424–627 nm) transition region and the area under the S22 (627–943 nm) transition region in the normalized (peak 727 nm) absorbance spectra was calculated. In Fig. 3b, this ratio and its relative change are plotted against the separation temperature.

At low temperatures, the M11/S22 ratio has a value of about 0.8. This value corresponds to a statistical SWCNT mixture without electronic type enrichment. For temperatures above 20 °C, the decrease in this ratio proofs an increase in the purity of the semiconducting SWCNT. For the separation temperature of 30 °C, a total decrease in M11/S22 ratio of about 30% is calculated. This value remains almost a constant for even higher temperatures. Nevertheless, the absolute absorbance (i.e. semiconducting SWCNT concentration) decreases.

The above described experiment proofs that WFC relies not only on protonation but also on selective colloidal aggregation that can be boosted at higher temperatures. Thus increasing the temperature during the centrifugation leads to an improved semiconductor enrichment. Nanoparticles and colloidal systems in general tend to lose their stability and agglomerate at higher temperatures^45^,^46^. The aggregation in homogeneous systems is often a result of van der Waals interaction between individual SWCNTs. For nanotubes, the van der Waals-London pair interaction energy depends on the diameter^47^. This suggests that the pH shift, observed in Fig. 2, could be resulting from the altered protonation and van der Waals-London interaction of larger diameter nanotubes.

In order to disentangle protonation from aggregation, we look at the individualization of SWCNTs in a neutral pH environment in the last part of this work. Using the PAE dispersant or sodium cholate hydrate, the temporal progress of the abundance of debundled SWCNTS under sonication was measured. The experiments were conducted at 2 different temperatures, 25 °C or 55 °C.

Four vials were set up containing 20 g/L aqueous dispersant namely the PAE polymer or sodium cholate hydrate (SCH) in combination with 5 g/L raw HiPco powder (5% SWCNTs). The vials were placed in a temperature controlled water bath. Cup Sonication (see methods) was applied and at selected times, 1 ml of the supernatant was collected and subsequently analysed by UV-vis-NIR spectroscopy. The amount of debundled SWCNTs correlates with the absolute absorbance^48^. To include the contribution of all chiralities, we evaluate the full area under the spectra.

**Figure 4:**
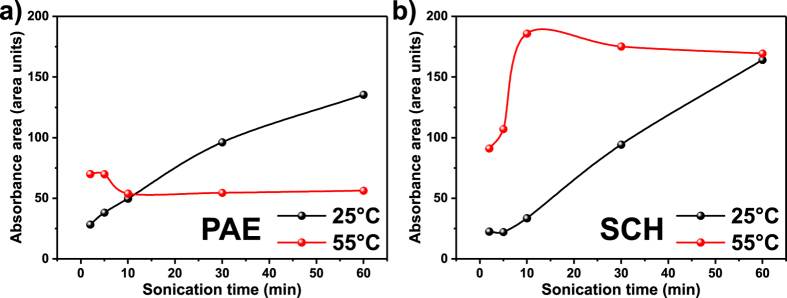
Individualization of SWCNTs in aqueous (**a**) PAE and (**b**) SCH as function of time and temperature using cup sonication. The area under UV-vis-NIR spectrum correlates with the amount of individualized SWCNTs.

In Fig. 4a, the influence of sonication time on the amount of dispersed nanotubes with the PAE at 25 °C and at 55 °C is shown. While at 25 °C the absorbance area increases linearly with time, at 55 °C no increase is observed. After 60 min of sonication the absorbance area roughly corresponds to one third of the absorbance area detected in the supernatant of the experiment conducted at 25 °C. This effect could be explained by a dehydration of the PEO moieties as reported elsewhere^49^,^50^. At 25 °C and using SCH, the same increase in absorbance area with sonication time as for PAE is detected as expected. Surprisingly, at 55 °C the increase of the amount of dispersed nanotubes with SCH is very steep. The supernatant is enriched with as much material as the maximum amount achieved at 25 °C but only in 10 minutes. However, at these relatively high temperatures the absolute amount of dispersed nanotubes remains relatively low. To visualize the dispersion with time photographs of the individual dispersions are shown for both temperature ranges in the Supporting Information.

This experiment proofs that even in neutral pH environment, PAE stabilized SWCNTS tend to agglomerate at higher temperatures since the amount of individual SWCNTs remains lower than at 25 °C in contrast to SCH. This implies that the mode of action behind the separation in WFC is a combination of protonation and selective aggregation.

## Conclusion

In summary, we have shown that the PAE dispersant has high affinity and no intrinsic selectivity with respect to adsorption onto nanostructured carbon surfaces. Subsequently, this polymeric dispersant is able to disperse SWCNTs of different diameters and stemming from different synthesis routes. This is a prerequisite for a diameter independent separation method of semiconducting SWCNTS. With a minor adjustment of the pH and using simply weak centrifugal fields different SWCNT raw materials can be in one single step purified. HiPco SWCNTs having a mean diameter of about 1 nm and Plasma Torch and Arc Discharge semiconducting SWCNT s with diameters ranging from 1.0 to 1.8 nm can be enriched. To our knowledge, water based diameter independent separation methods with high upscaling potential and no use of a pre-purification step are not yet reported.

Furthermore, the influence of temperature while dispersing the carbon material and during separation was also investigated. Increasing the temperature while dispersing reduces the ability to de-bundle the nanotubes. Increasing the temperature during the centrifugation leads to an improved semiconductor enrichment. That implies that the separation is mainly driven by the protonation and selective aggregation of PAE stabilized metallic nanotubes. This unique mode of action enables a diameter independent separation method, i.e. wide dynamic range method, to enrich mixtures of semiconducting SWCNTS.

## Methods

### Preparation of SWCNT – dispersions

HiPco SWCNTs were purchased from NanoIntegris (Batch# HS 28030 with 5 wt% SWCNTs, Batch# P2-772 with 80 wt% SWCNTs), Plasma SWCNTs were purchased from Raymor Nanotech (RN-020) and Arc discharge SWCNTs were purchased from Carbon Solutions Inc. (P2). Out of Gaussian fits to the absorbance of the raw dispersions we deduced the mean diameters and the diameter distribution as follows^51^,^52^: HiPco SWCNTs (1.17 nm ± 0.13 nm), Plasma SWCNTs (1.30 nm ± 0.10 nm) and Arc discharge SWCNTs (1.44 nm ± 0.12 nm). The as-produced SWCNTs were dispersed in an aqueous solution containing 2 wt% of PAE by horn sonication for 1 h at 100% amplitude (Dr. Hielscher UP200s) starting with 0.5 wt% of raw nanotube material in a total of 50 g of dispersion. During sonication the vials were placed in ice-cooled water bath.

### Adsorption of PAE on nanotubes

The as-produced HiPco SWCNTs were dispersed in an aqueous solution containing 0.1–5 wt% of PAE or Pluronic F68 or F108 by horn sonication for 40 h at 100% amplitude (Dr. Hielscher UP200s) starting with 2.5 wt% of raw nanotube material in a total of 30 g of dispersion. During sonication the material was placed in ice-cooled water bath.

The dispersions were centrifuged 5 times for 4 h at 250.000 × *g* after each run the complete liquid phase was extracted, homogenized and submitted to the next run. After each run a nanotube pellet stuck to the bottom of the centrifugation tube and was discarded this way. Via absorbance (PAE) or refractive index measurements the concentration of PAE in the supernatant was calculated.

Pluronic and PAE solutions of known concentrations were used and treated equally to the nanotube dispersion to calibrate the extinction coefficient at 278 nm or the refractive indices (See Supporting Information).

### Centrifugation process

For centrifugation, a water based solution of sodium polytungstate (SPT) (TC Tungsten Compounds) 25.5 wt% also containing 2 wt% of PAE was prepared. The pH-value of this SPT column was controlled by adding small aliquots of 1 M HCl. The centrifugation vessel was loaded with 4.2 ml of SPT (pH 2.0) and 0.3 ml of the nanotube dispersion (pH 4) on top. Centrifugation (Beckman Coulter Optima XL) was performed applying a centrifugal field of approximately 10,000 × *g* for 18 h to separate nanotubes in a Beckman Coulter SW 60Ti rotor.

### Temperature dependent centrifugation

The centrifugation conditions were kept as described above. After inserting the rotor into the centrifuge, the temperature was allowed to settle for 0.5–4 h until equilibrium was reached (±0.1 °C).

### Post-treatment of the separated SWCNTs and re-dispersion into nanotube inks

The extracted semiconducting SWCNT fractions that still contained SPT and the PAE polymer were pelletized by centrifugation at 40.000 × *g* for 17 h after dilution with DI-water. The supernatant was removed and the pellet was swirled up by refilling the centrifugation vessel with DI-water. This manoeuvre was repeated 2 times. Afterwards the pellet was collected in an aqueous solution of 1 wt% of Sodium deoxycholate (Alfa-Aesar) (pH 7.2). Tip-sonication for 15 min was used to redisperse the SWCNTs.

### Temperature dependent SWCNT – dispersions

The Branson Digital Sonifier 450 was equipped with a sonication chamber so that the sonication power from the horn is directed into a water flushable chamber (cup). The sonicator was inverted and the sonication cup is placed on top of the horn of the sonicator. The cup itself is attached to a cooling water supply. Via a three pipe system the water flow can be adjusted so that a constant temperature can be maintained during sonication.

### Characterization

Optical analysis was performed by recording the absorbance spectra of post-treated samples with the Perkin Elmer UV-vis-NIR Spectrometer Lambda 250 with 1 cm quartz cuvettes.

### Field-Flow Fractionation

The AF4 was performed using the separation system Eclipse AF4 from Wyatt Technology. The PAE sample was concentrated as 0.3 wt% (3 g/L) aqueous solution with 0.05 M NaNO_3_ and injected into the channel equipped with a 5 k Dalton RC-membrane (Millipore). The detector flow was 0.5 ml/min. During the measurement an exponentially decreasing cross flow was used. The cross flow decreased from 4 ml/min to 0.1 ml/min over a period of 35 min. The separation system was equipped with a light scattering system (DAWN Helios-II) and a refractive index measurement system (Optilab T-rEX) both from Wyatt Technologies.

## Additional Information

**How to cite this article:** Reis, W. G. *et al*. Wide dynamic range enrichment method of semiconducting single-walled carbon nanotubes with weak field centrifugation. *Sci. Rep.* **7**, 44812; doi: 10.1038/srep44812 (2017).

**Publisher's note:** Springer Nature remains neutral with regard to jurisdictional claims in published maps and institutional affiliations.

## Supplementary Material

Supplementary Information
