# Supplementary material for: Wide dynamic range enrichment method of semiconducting single-walled carbon nanotubes with weak field centrifugation
Source: Sci Rep. 2017 Mar 20;7:44812. doi: 10.1038/srep44812 (PMC5357843; doi:10.1038/srep44812)
Supplement: Supplementary Information [file srep44812-s1.pdf]

Supporting Information

Wide dynamic range enrichment method of semiconducting single-walled carbon nanotubes with weak field centrifugation

Wieland G. Reis[1], Željko Tomović[1,*], R. Thomas Weitz[2], Ralph Krupke[3] and Jules Mikhael[4,*]

[1] Carbon Materials Innovation Center (CMIC), BASF SE, 67056 Ludwigshafen, Germany

[2] Physics of Nanosystems, Physics Department, NanoSystems Initiative Munich and Center for NanoScience (CeNS) Ludwig Maximilians Universität München, Amalienstrasse 54, 80799 Munich (Germany)

[3] Department of Materials and Earth Sciences, Technische Universität Darmstadt, 64287 Darmstadt, Germany

[4] Material Physics Research, BASF SE, 67056 Ludwigshafen, Germany

* Corresponding Authors E-mail: zeljko.tomovic@basf.com, jules.mikhael@basf.com

Chemical structure of PAE polymer

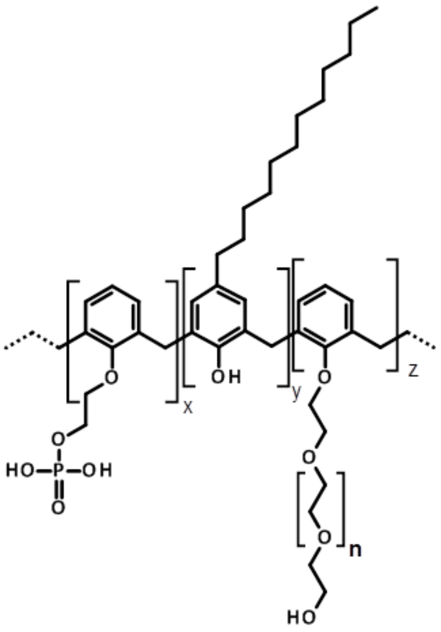

x=10
y=2
z=1
n=30

Figure S1. Idealized structure of the PAE dispersant. The average composition is denoted.

Absorbance spectra and refractive index calibration for polymer adsorption calculations

The absorbance was calibrated for different concentrations to determine the adsorbed polymer concentration via absorbance measurements. The calibration concentrations of the aqueous PAE polymer were set up in concentrations between 1 and 30 g/L. They were diluted by a factor of 100 to have the peak absorbance (278 nm) of the polymer in the linear regime of the detector system (Perkin Elmer 35). In Figure S2a, the UV-vis-NIR absorbance spectra of the polymeric solutions are shown. In Figure S2b, the absorbance at 278 nm was plotted over the concentration. A linear fit was used to describe the data.

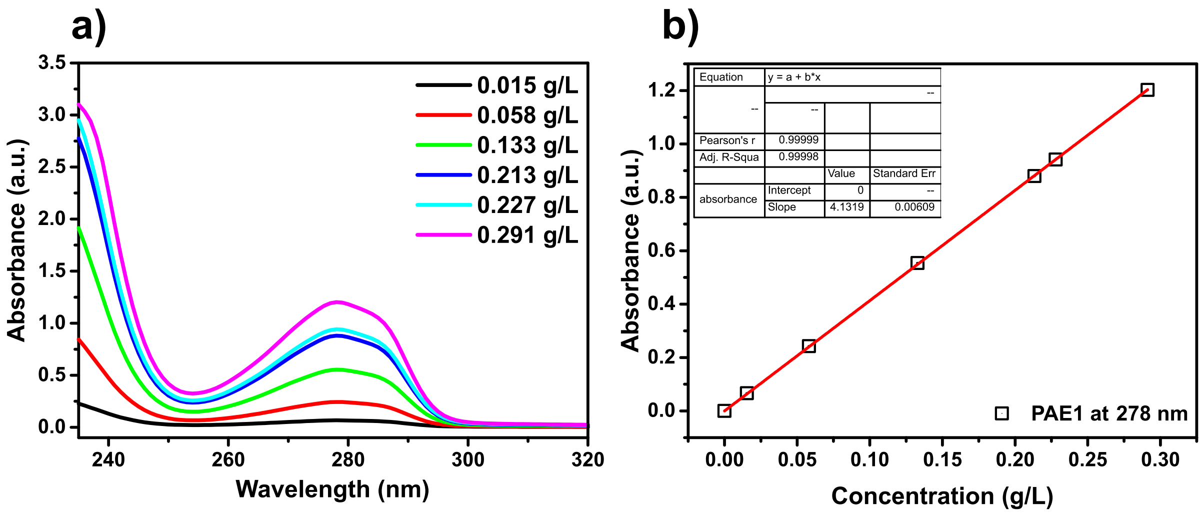

Figure S2. In a) absorbance spectra of calibration solutions of the PAE polymer are shown. In b) the peak absorbance values are used to calibrate the relation between concentration and absorbance at 278 nm.

The absorbance of the supernatants (obtained as described in the main text) was used to calculate the corresponding free and adsorbed polymer concentrations. The adsorbed polymer concentration is calculated via the difference between free polymer and initially polymer concentration. The measured and rounded calculated values are summarized in Table S1.

Measured Absorbance (diluted) (a.u.)	Concentration (diluted) (g/L)	Concentration (g/L)	Input Concentration (g/L)	Adsorbed Polymer Concentration (g/L)
0.036	0.00883	1.01	01.04	0.03
0.181	0.04374	4.23	05.02	0.79
0.667	0.16142	13.79	14.96	1.18
0.743	0.17982	18.81	19.95	1.14
1.232	0.29822	23.48	24.97	1.48
1.163	0.28157	28.48	30.00	1.52
1.171	0.28334	49.16	49.98	0.82

The calibration concentrations of the aqueous Pluronic F68 and F108 polymers were set up between 1 and 50 g/L. Due to a lack of peaks in the UV-vis-NIR absorbance spectra, the refractive index was measured and plotted versus concentration in Figure S3.

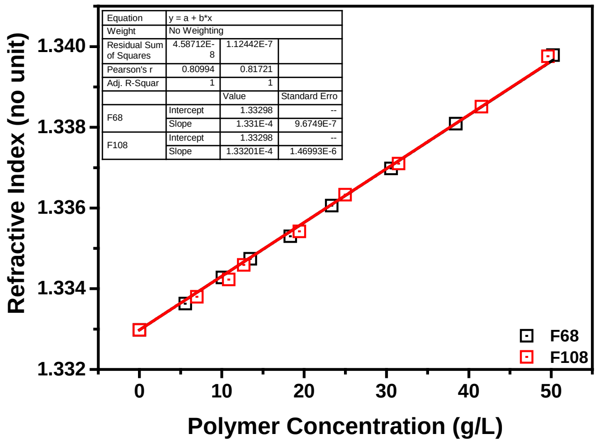

Figure S3. The refractive index calibration of Pluronic F68 and Pluronic F108.

The refractive indices of the supernatants (obtained as described in the main text) were used to calculate the corresponding adsorbed polymer concentrations. The adsorbed polymer concentration was calculated via the difference between free polymer and initially polymer concentration. The measured and calculated values of the measurements are summarized in Table S2.

Polymer	Measured Refractive Index Supernatant	Free Polymer Concentration (g/L)	Initial Polymer Concentration (g/L)	Adsorbed Polymer Concentration (g/L)
F68	1.33688	29.31	29.98	0.67
F108	1.33691	29.51	29.95	0.44

Neutral DGU of Stable Dispersions

Dispersions created with the three polymeric dispersants (PAE, F68, F108) were analysed regarding their SWCNT content. A neutral DGU step at 40,000 x *g* was applied for 14 h to sort the HiPco SWCNT material from the amorphous carbon. The aqueous density material was Sodium Polytungstate (SPT) at 26 wt%. The separated nanomaterial (supernatant) was extracted, diluted and pelletized by multiple centrifugation runs at 40,000 x *g*. The obtained pellets were dispersed in 0.5 wt% sodium deoxycholate for UV-vis-NIR absorbance measurements. These measurements were shown in the main text (Figure 1).

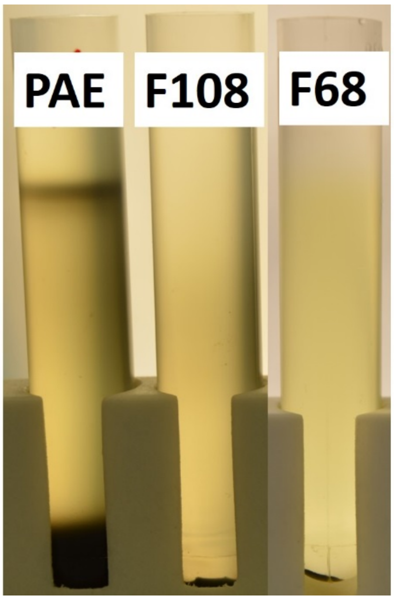

Figure S4. DGU of stable polymeric dispersions. From left to right, 1 ml of a raw HiPco dispersion with 2 wt% aqueous PAE, Pluronic F108 and Pluronic F68 were centrifuged on a neutral column (3.4 ml) of aqueous SPT (26 wt%) at 40,000 x *g* for 14 h.

Dispersions with different PAE concentrations

The supernatant of centrifuged (250.000 x *g*, 30 min) HiPco dispersions with PAE concentrations between 10^{-3} and 10 g/L were analysed by UV-vis-NIR measurements. The corresponding spectra are shown in Figure S5.

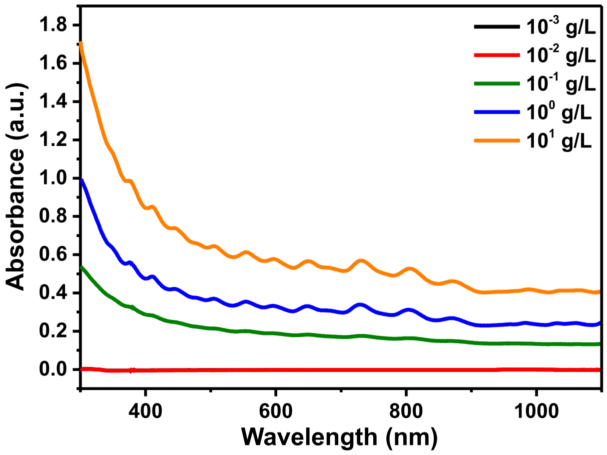

Figure S5. UV-vis-NIR absorbance spectra of dispersions created with increasing amounts of PAE polymer as dispersant.

Dispersions of PAE with different SWCNT sorts

The different SWCNT sorts (HiPco, Arc Discharge and Plasma Torch) were dispersed with aqueous PAE concentrations of 2 wt%. Each dispersion was analysed by UV-vis-NIR measurements. The corresponding spectra are shown in Figure S6.

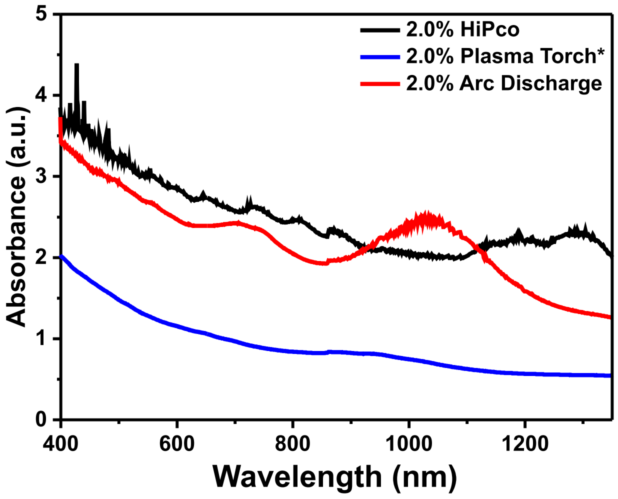

Figure S6. UV-vis-NIR absorbance spectra of dispersions created the PAE polymer as dispersant and different SWCNT materials. SWCNTs synthesized via the HiPco, Plasma torch and Arc Discharge process can be dispersed using the PAE polymer.

Spectra of SWCNTs from temperature dependent WFC separation

The temperature dependent WFC at acidic conditions lead to the isolation of SWCNT fractions. The fractions were extracted, diluted, pelletized and re-dispersed in 0.5 wt% sodium deoxycholate (DOC). Each fraction was subsequently analysed using UV-vis-NIR spectroscopy. The resulting spectra are displayed in Figure S7.

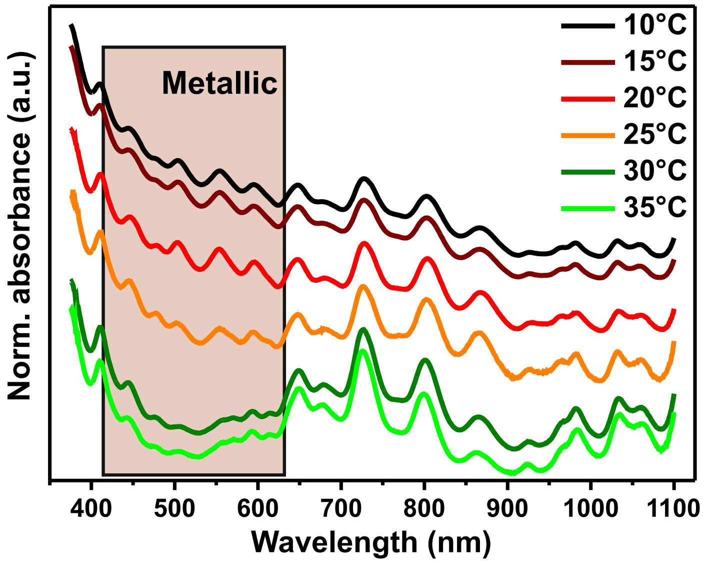

Figure S7. UV-vis-NIR absorbance spectra of extracted, diluted, pelletized and re-dispersed SWCNTs from temperature dependent WFC separation are shown. The absorbance in the metallic transition region decreases with increasing temperature. The spectra were offset for clarity.

Temperature dependent dispersing of SWCNTs

Two different dispersant sodium cholate hydrate (SCH) and the PAE polymer were used to disperse SWCNTs at different temperatures. The dispersions were photographed after different sonication time intervals (2 min, 5 min, 10 min, 30 min and 60 min). The supernatants were analysed via UV-vis-NIR absorbance measurements. The photographs of the individual dispersions are shown in Figure S8.

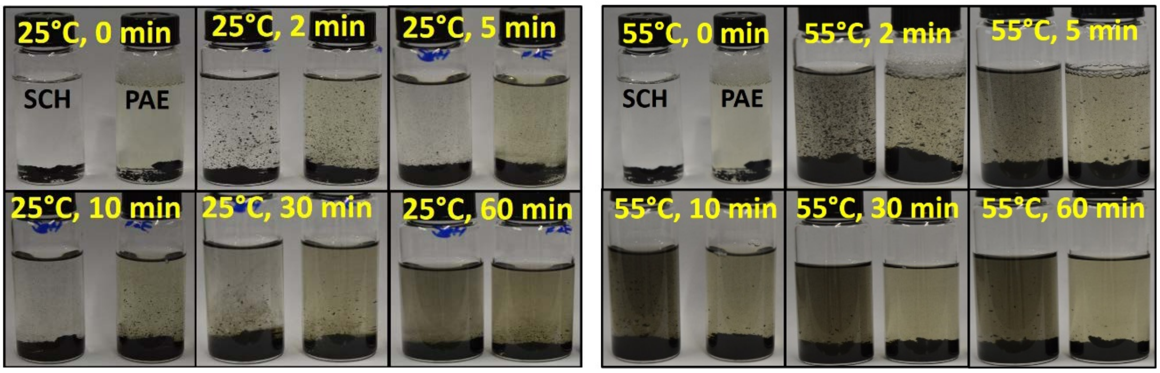

Figure S8. Photographs of temperature dependent dispersions using dispersants PAE and sodium cholate (SCH). The 0 min photograph from the 25°C series was used to fill the gap in the 55°C series.

Diameter distribution in SWCNT dispersions

Background correction was performed by subtracting a straight line background in the S11 transition area of the HiPco raw dispersion or in the S22 transition region of the Plasma and Arc Discharge SWCNT dispersions. After subtraction of the background, Gaussian fits were applied to the respective transition areas of each UV-vis-NIR spectrum. From the Gaussian fits the diameter distribution was calculated via

$$S11 = \frac{2\gamma_0\, a_0}{d},\ \ S22 = \frac{4\gamma_0\, a_0}{d},$$

where a_0=0.142 nm and γ_0 = 3.0 eV [1, 2].

TEM image of separated semiconducting HiPco SWCNTs

A TEM image in Figure S9 shows the drop casted dispersion of separated semiconducting SWCNTs. The image shows solely nanotubes indicating the exclusion of amorphous carbon and other impurities by the weak field centrifugation (WFC) separation method.

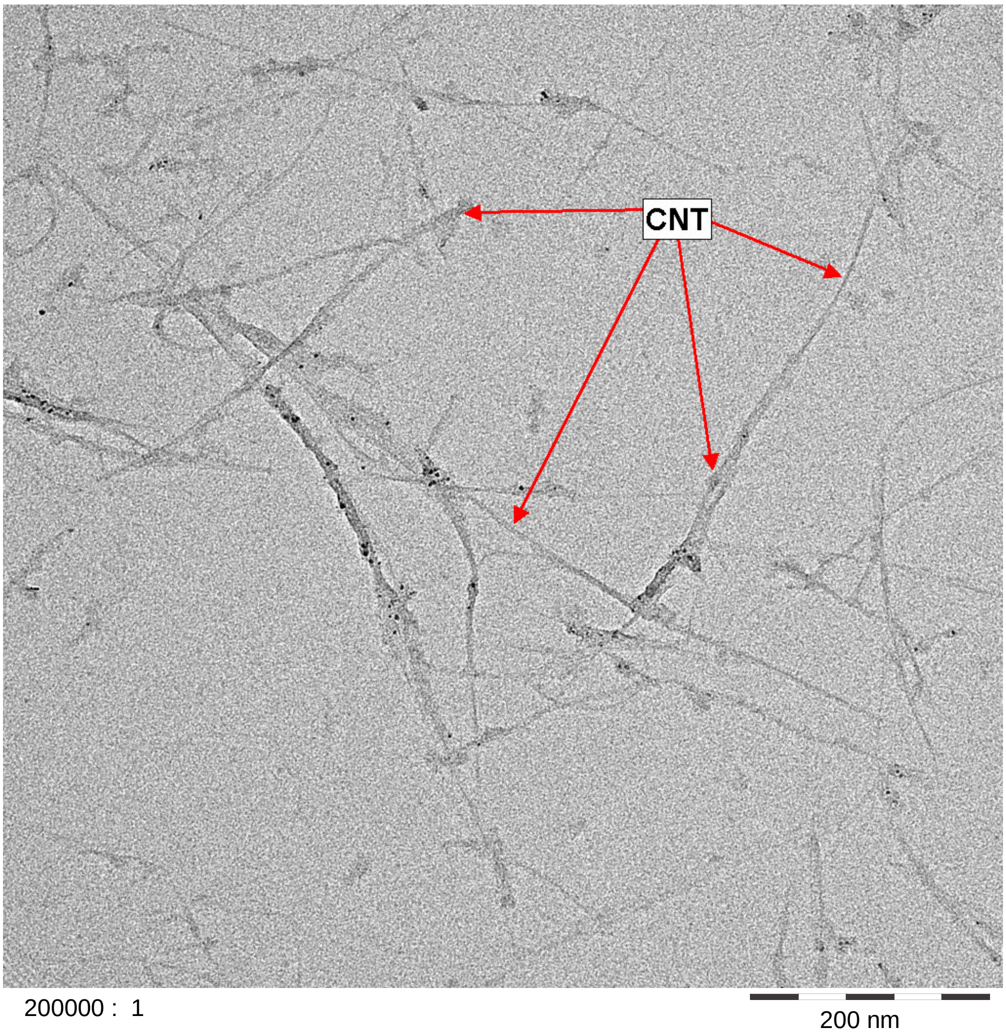

Figure S9. Transmission electron microscopy image of the separated semiconducting SWCNTs.

References

1. Liu, X. *et al.* Detailed analysis of the mean diameter and diameter distribution of single-wall carbon nanotubes from their optical response. *Physical Review B* **66,** 045411 (2002).
2. Chakravarty, A. & Biswas, S. K. Enrichment of metallic single-walled carbon nanotubes with simultaneous purification by nitric acid treatment. *Fullerenes, Nanotubes and Carbon Nanostructures* **23,** 542-548 (2015).
